# *Brucea javanica* Oil Emulsion Promotes Autophagy in Ovarian Cancer Cells Through the miR-8485/LAMTOR3/mTOR/ATG13 Signaling Axis

**DOI:** 10.3389/fphar.2022.935155

**Published:** 2022-07-25

**Authors:** Yihan Wang, Bocen Chen, Man Xiao, Xiaoli Wang, Yunhua Peng

**Affiliations:** ^1^ The Second Clinical College, Hainan Medical University, Haikou, China; ^2^ Key Laboratory of Molecular Biology, School of Basic Medicine and Sciences, Hainan Medical University, Haikou, China; ^3^ Hainan Women and Children’s Medical Center, Haikou, China; ^4^ The First Affiliated Hospital of Hainan Medical University, Haikou, China

**Keywords:** *Brucea javanica* Oil Emulsion, miR-8485, ovarian cancer, autophagy, traditional Chinese medicine

## Abstract

**Background:** Ovarian cancer is a common malignant tumor of the female reproductive tract, with the highest mortality rate. At present, no effective approaches to improve the survival rate exist. *B. javanica* Oil Emulsion (BJOE), an extract from *B. javanica (L.) Merr.* [Simaroubaceae], exhibits antitumor effects and can increase the sensitivity of radiotherapy and chemotherapy in many types of cancers. MiR-8485, a discovered miRNA, has been shown to be involved in the occurrence and development of tumors. The purpose of this study was to investigate the effect of BJOE on the regulation of mammalian rapamycin target protein (mTOR) autophagy signal pathway and related autophagy factors on ovarian cancer cells through miR-8485.

**Methods:** The main chemical constituents of BJOE were determined by UHPLC-MS/MS. Detection of miR-8485 expression in ovarian cancer cells treated with BJOE by quantitative reverse transcription polymerase chain reaction (qRT-PCR). CCK8 experiment and flow cytometry were used to observe the effects of BJOE and overexpression of miR-8485 on cell proliferation and apoptosis. Then, monodansylcadaverine (MDC) fluorescence staining was used to observe the changes of autophagy vesicles before and after the effect of BJOE and overexpressed miR-8485 on cancer cells. Next, the binding sites between miR8485 and mammalian rapamycin target protein activator 3 (LAMTOR3) were detected by double luciferase reporter assay. Furthermore, qRT-PCR and Western blot experiments were used to explore the changes of autophagy-related factors LAMTOR3, mTOR and autophagy-related 13 (ATG13), and microtubule associated protein 1 light chain 3 beta (LC3-Ⅱ) after BJOE and overexpression of miR-8485, in addition to autophagy inhibitor (3-MA) for rescue experiment verification.

**Results:** The qRT-PCR results showed that the expression of miR-8485 increased after BJOE treatment in the SKOV3 cell. The CCK8 assay and flow cytometry analysis revealed that both BJOE and miR-8485 overexpression inhibited the proliferation and promoted the apoptosis of the SKOV3 cell. MDC fluorescence staining showed that BJOE and miR-8485 overexpression led to a significant increase in autophagy vesicles in the SKOV3 cell. Double luciferase reporter assay confirmed the existence of binding sites between miR8485 and LAMTOR3. The results of qRT-PCR and Western blot showed that BJOE and overexpressed miR-8485 downregulated the expression of LAMTOR3 and mTOR and up-regulated the expression of ATG13 and LC3-Ⅱ.

**Conclusion:** 1) MiR-8485 may be the key factor of BJOE in promoting autophagy and apoptosis and inhibiting cell proliferation of ovarian cancer cells; 2) BJOE may play an antitumor role by regulating LAMTOR3/mTOR/ATG13 signaling axis through miR-8485 to promote autophagy in ovarian cancer cells.

## Introduction

According to the latest statistics, 19,880 new cases of ovarian cancer are expected in 2022, accounting for 1.0% of all new cancer cases, and 12,810 deaths from ovarian cancer are expected in 2022, accounting for 2.1% of all cancer deaths. By 2018, the 5-year relative survival rate of ovarian cancer patients was 49.7%, and that of non-Hispanic Asian/Pacific Islander was 56.5% (https://seer.cancer.gov/statfacts/html/-ovary.html). A lack of sensitive and specific tumor markers exists, and ovarian cancer is prone to pelvic invasion and metastasis (Agarwal and Kaye, 2005). In current times, the major treatment of this cancer is surgically combined with chemotherapy after operation, and relapsing after operation is easy (Agarwal and Kaye, 2005; Moore et al., 2018; Coleman et al., 2019; Akilli et al., 2022). The use of chemotherapy drugs often lead to serious side effects and multidrug resistance (Agarwal and Kaye, 2005; Moore et al., 2018; Coleman et al., 2019; Li and Xia, 2020; Xu et al., 2021; Akilli et al., 2022). With the progress of the research on the treatment of ovarian cancer, the application of traditional Chinese medicine has become increasingly prominent (Xu et al., 2021). Traditional Chinese medicine not only demonstrates the advantages of many therapeutic targets, low resistance, definite curative effect, few side effects, low cost, and reducing the toxic and side effects of chemotherapeutic drugs (Li and Xia, 2020). Also, it can prevent cell growth, invasion, and apoptosis of ovarian cancer cells (Agarwal and Kaye, 2005). Thus, to develop novel therapeutic intervention for this malignant tumor, exploring mechanisms by which traditional Chinese medicine protects against ovarian cancer is essential.

*B. javanica* (*L*.) *Merr.* is the extract of the mature fruit of *B. javanica* (*L.*) *Merr.* [Simaroubaceae], a traditional Chinese medicine plant of bitter wood family (Fang, 2005; Yang et al., 2010). Researchers found that *B. javanica (L.) Merr.* demonstrates high affinity to cancer cells. *B. javanica* Oil Emulsion (BJOE) is extracted from the dried and ripe fruit of *B. javanica (L.) Merr.* [Simaroubaceae] (Yang et al., 2010).

In this experiment, the clinical anticancer preparation *B. javanica* Oil Emulsion injection was used. The main components included refined *B. javanica* oil. The excipients were refined soybean phospholipids, glycerol, and injection water, which were milky white uniform emulsion. Brucea javanica oil emulsion injection was used for intravenous infusion and was easy to transport and absorb blood. BJOE exhibits a nonspecific antitumor effect in cell cycle (Yang et al., 2010). The oleic acid and linoleic, the components of BJOE, can inhibit the oxygen uptake of cancer cells to induce hypoxic death and inhibit the DNA synthesis of tumor cells; the surface activity of fatty acids is toxic to cancer cells, and excessive accumulation can lead to the rupture of cancer cell membrane; the emulsion can make the drug more directional distribution, so that BJOE demonstrates a higher concentration in ovarian cancer cells, in order to improve the anticancer effect (Fang, 2005; Yang et al., 2010). At present, the main mechanisms of BJOE in tumors include inhibiting tumor proliferation, reversing drug resistance, increasing the efficacy of chemotherapy, reducing the toxic and side effects of drugs on the body, and preventing tumor progression (Zhao et al., 2015a; Zhao et al., 2015b; Sun, 2009; Chen et al., 2009; Xu et al., 2008; Chen et al., 2015; Tang et al., 2014). Studies showed that centrosome amplification promotes tumor cell survival and proliferation, epithelial–mesenchymal transition promotes tumor cell migration and invasion, and autophagy can participate in tumorigenesis and inhibit tumor progression (Mittal et al., 2021; Long et al., 2022; Paskeh et al., 2022). Autophagy is a self-phagocytosis and self-protection mechanism of cells. Increasing evidence exists that autophagy is not only closely related to tumor progression and multidrug resistance, but it also affects the expression of many oncogenes and tumor suppressor genes (Li et al., 2017; Chen et al., 2019; Poillet-Perez and White, 2019). The effect of BJOE on tumor autophagy occurred only in human non-small cell lung cancer and colon cancer cell (Yan et al., 2015; Zhu and Pan, 2018).

A variety of studies showed that Phosphatidylinositol 3-kinase (PI3K) and Serine/threonine kinase 1 (AKT) are activated in ovarian cancer cells, which positively regulate their downstream genes mTOR, and inhibit cell autophagy, proliferation, apoptosis, and metabolic function (Mabuchi et al., 2015). Thus, targeting the PI3K-AKT-mTOR, LKB1-AMPK-mTOR, MAPK, and other signal pathways has become the direction of ovarian cancer treatment (Mabuchi et al., 2015; Peng et al., 2021; Takahashi et al., 2021; Blagden, 2022). Mammalian rapamycin target protein (mTOR) is a key target in regulating the autophagic process, and many agents can affect its expression. Mammalian rapamycin target protein activator 3 (LAMTOR3) alias MP1, located on human chromosome 4q22.3, is a protein-coding gene that encodes scaffold proteins and is one of the Ragulator complexes (Sun and Xu, 2012). It binds to and activates Rag GTPases on lysosomes by lipid modification and recruits mTORC1 until lysosomes are activated. Also, activated mTORC1 can simultaneously acidify the homologous protein unc-51 like autophagy activating kinase 1 of autophagy-related 13 (ATG13) and autophagy-related 1 (ATG1) in mammals to inhibit autophagy (Sun and Xu, 2012). Our group’s early bioinformatics analysis showed that the pathway of *B. javanica (L.) Merr.* [Simaroubaceae] in ovarian cancer cells was also mainly concentrated in 14 signal pathways, such as mTOR, vascular endothelial growth factor (VEGF), and mitogen activated kinase-like protein (MAPK) (Peng et al., 2019). Cell autophagy can be affected by many factors. Methylation of autophagy-related genes affects the growth of tumor cells by regulating autophagy (Lattanzio and Nigro, 2015). To activate autophagy and inhibit tumor occurrence and migration, histone deacetylase inhibitors act on autophagy-related genes and signal pathways, while miRNA can regulate the expression of autophagy-related genes and affect tumor progression (Li and Wan, 2012; Ngamphaiboon et al., 2015). Among them, the study of miRNA on autophagy of ovarian cancer cells has been a popular topic in recent years, mainly involving miR-126-5p and miR-129 (Bi et al., 2021; Zhang et al., 2021).

The miR-8485 detected by dbDEMC (Human Cancer differential expression MiRNAs Database: https://www.biosino.org/dbDEMC/index) showed a tendency of low expression in the blood of different cancers, such as ovarian cancer. However, the existing tumor research focused on oral and laryngeal squamous cell carcinoma, and the mechanism of miR-8485 in ovarian cancer has not been reported. miR-8485 (miR-NID1) is processed from intron 5 of human neurofilament 1 (NRXN1) and inhibits NRXN1 expression by binding to TAR DNA binding protein (TDP-43) (Fan et al., 2014). This study aimed to explore whether BJOE participated in the development of ovarian cancer, by affected autophagy mediated by miR-8485 regulating LAMTOR3/mTOR/ATG13 signaling axis, thereby providing feasible ideas for targeted therapy of ovarian cancer and scientific basis for the rational use of *B. javanica (L.) Merr.* [Simaroubaceae] for the diagnosis and treatment of tumors.

## Material and Methods

### Reagents and Materials

Human ovarian cancer cell line SKOV3 was purchased from Shanghai Hongshun Biotechnology Co., Ltd. (Shanghai, China). Human ovarian epithelial (IOSE80) cells were provided by Shanghai Zeye Biotechnology Co., Ltd. (Shanghai, China). SKOV3 and IOSE80 cells were cultured in McCoy’s 5A and Dulbecco's Modified Eagle Medium containing 10% fetal bovine serum and 1% penicillin/streptomycin, respectively. These cells were placed in a 5% CO_2_ cell incubator at 37°C for microscopic observation. LAMTOR3 and ATG13 were purchased from Proteintech Group, Inc., United States. Next, antibodies against mTOR and LC3B were purchased from abcam Company, United States. Also, RNA extraction kit was purchased from Shanghai Plomag Biological products Co., Ltd. (Shanghai, China). A reverse transcription kit and real-time fluorescent quantitative polymerase chain reaction (PCR) kit were purchased from Shanghai Yisheng Biotechnology Co., Ltd. (Shanghai, China). miRNA mimics and miRNA NC mimics purchased from Ruibo (Guangzhou, China). MiRNA lentivirus was purchased from Shanghai Hanbio Biotechnology Co., Ltd. (Shanghai, China). 3-methyladenine (3-MA) was purchased from MedChemExpress, United States. *B. javanica* Oil Emulsion injection was purchased from Shenyang Daleiyun Pharmaceutical Co., Ltd. (Shenyang, China).

### Ultra-High Performance Liquid Chromatography–Quadrupole Time-Of-Flight–Mass Spectrometry Analysis

Samples are thawed on ice; take the supernatant 500 μl in EP tube, add 10 μl mixed internal standard; vortex 30 s, ice bath ultrasonic 15 min; sample 4°C, 12,000 rpm [centrifugal force 13,800 (×g), radius 8.6 cm] centrifugal 15 min; 5. Carefully filter the supernatant with 0.22 μm microporous membrane; the supernatant after membrane was centrifuged at 4°C, 12,000 rpm [centrifugal force 13,800 (×g), radius 8.6 cm] for 15 min; the supernatant was carefully filtered by 0.22 μm microporous membrane and stored at −80°C until tested. Vanquish (Thermo Fisher Scientific Ultra Performance Liquid Control) was analyzed according to the mobile phase parameters in the table below. Next, the chromatographic column used was UPLC BEH C18 column (1.7 μm × 2.1 × 100 mm) purchased from Waters. The injection volume was 5 μl. Note the following: The time unit is min, the flow rate unit is μl/min. Next, 0.1% formic acid is added in A and B phases. An Orbitrap Exploris 120 mass spectrometer coupled with an Xcalibur software was employed to obtain the MS and MS/MS data based on the information dependent acquisition mode. These experiments were performed by Shanghai Biotree Biomedical Technology Co., Ltd. (Shanghai, China).

### Cell Counting Kit-8 Assay

Cells were centrifuged and counted, and 96-well plates were laid. According to the drug concentration, it was mixed with the medium in proportion. Then, the drug-containing medium was added to the cells for further culture (field preparation when in use). The blank group, mimic NC group, mimic group, and *B. javanica* Oil Emulsion drug gradient group were set. After 24 h culture in the incubator, the mimic NC group and mimic group were replaced with antibiotic-free medium, and the transfer solution configured with the basic medium by Lipo6000 was added. The complete medium was changed 6 hours after transfection. After 24 h culture in the incubator, 10 μl CCK8 solution was added to the fresh medium of each well, and the absorbance at 450 nm was measured by an enzyme-labeled instrument. The absorbance was measured once every 1 h and 4 times.

### Real-Time Quantitative Polymerase Chain Reaction Analysis

Total RNA was extracted by RNA extraction kit, mRNA was reverse transcribed by reverse transcription kit, and miRNA was reverse transcribed by miRNA first-strand cDNA synthesis (stem loop). All operations are performed according to the kit instructions. Detection of gene expression by q225 fluorescence quantitative PCR and the reaction conditions were carried out according to the operation instructions of the fluorescence quantitative PCR kit (SYBR GreenMix, Roche). Thermal cycle parameters are as follows: 95°C for 300 s, then 95°C for 10 s, 60°C for 30 s, a total of 40 cycles. Three independent replicates were set for each reaction of quantitative PCR. Next, U6 was used as an internal control for miRNA, with glyceraldehyde-3-phosphate dehydrogenase for coding genes. Data analysis used the 2^−Δ^Ct method, ^Δ^Ct = experimental group (Ct target gene-Ct reference)-control group (Ct target gene-Ct reference). The amplified sequences of each gene and its internal reference are listed in Table 1.

**TABLE 1:** Primer sequences used in quantitative reverse transcription polymerase chain reaction.

Name of primer	Sequences
U6-F	5′-AGA​GAA​GAT​TAG​CAT​GGC​CCC​TG-3′
U6-R	5′-CAG​TGC​AGG​GTC​CGA​GGT-3′
miR-8485-F	5′-CGC​GCA​CAC​ACA​CAC​ACA​C-3′
miR-8485-R	5′-AGT​GCA​GGG​TCC​GAG​GTA​TT-3′
GAPDH-F	5′-GTG​AAG​GTC​GGA​GTC​AAC​G-3′
GAPDH-R	5′-TGA​GGT​CAA​TGA​AGG​GGT​C-3′
LAMTOR3-F	5′-ATG​GCG​GAT​GAC​CTA​AAG​CG-3′
LAMTOR3-R	5′-ATG​GAG​CCC​TTC​AAC​ACT​TGG-3′
mTOR-F	5′-AAG​ATG​CTT​GGA​ACC​GGA​CC-3′
mTOR-R	5′-CTG​GTT​TCC​TCA​TTC​CGG​CT-3′
ATG13-F	5′-AGT​CAA​GTG​CCT​AGC​CTC​AC-3′
ATG13-R	5′-GCC​TGC​TCC​AAT​CCT​CAG​AA-3′
LC3B-F	5′-AGA​ATG​ATA​CCA​GGG​TGA​GAA​GG-3′
LC3B-R	5′-TCT​CAC​CCT​CAT​ACA​CCT​CTG​A-3′

### Luciferase Report Assay

The wild type (Wt) or mutant (Mut) LAMTOR3 fragment was linked to pmirGLO vector. Next, the mutant gene sequence was produced by Tsingke Biotechnology Co., Ltd. SKOV3 cells were seeded in 24-well plates (1 × 10^5^ cells/well) and then co-transfected with Wt or Mut vector (1 µg) with miR-8485 mimic (10 nM) or NC mimic (10 nM) by using lip6000. After 24 h, the luciferase activity was detected by dual luciferase reporter gene detection system (Tsingke Biotechnology Co., Ltd.). The relative luciferase activity is calculated by the ratio of firefly luciferase activity to reninase activity.

### Cell Transfection Assay

After the cells were counted by centrifugation, a 6-well plate was laid, and the culture plate was pre-cultured in the incubator for 24 h. Mimic NC, simulated miR-8485, and Lipo6000 were added to the culture plate for plasmid transfection. Six hours later, the complete medium was replaced, and the transient state was observed 24 after 24 h. On the other hand, lentivirus transfection was carried out in the miR-8485 overexpression group and miR-8485 non-loaded group, and stable cell lines with high expression of miR-8485 were screened by puromycin in the later stage.

### Cell Apoptosis Assay

After the cells were counted by centrifugation, a 6-well plate was laid, and the culture plate was pre-cultured in the incubator for 24 h. Mimic NC, simulated miR-8485, and Lipo6000 were added to the culture plate for plasmid transfection and then replaced with complete medium after 6 h. After 24 h, the cells were digested and collected with trypsin without ethylenediamine tetraacetic acid and washed twice with precooled phosphate-buffered saline (PBS). According to the instructions provided by the manufacturer, the cells were stained with AnnexinV-FITC and propidium iodide (PI) dyes, respectively, and then analyzed by flow cytometry. This method can distinguish early apoptotic cells (Annexin-V/FITC+/PI−) from late apoptotic cells (Annexin-V/FITC+/PI+). In this experiment, these two parts of cells were counted as apoptotic cells.

### Western Blot Analysis

Extraction of total protein from human meningeal cell lysate occurred with 1 mlRIPA buffer containing 10 μl phenylmethylsulfonyl fluoride and 20 μl phosphorylase inhibitors. The same amount of protein (40 μg) was loaded into 10% sodium dodecyl sulfate-polyacrylamide gel for separation. The membrane and the first antibody were incubated overnight at 4°C and then incubated with antirabbit second antibody at room temperature for 1 h. Visual imprinting using an enhanced chemiluminescence detection kit (ECL, Thermo Science, United States). Image capture used G·BLOT automatic chemiluminescence image analysis system (G·BLOT, Shanghai, China) and analysis using Touch Image.

### Mono-Dansylcadaverine Staining Experiment

The cells were centrifuged and counted, and 6-well plates were placed. The plates were pre-cultured in the incubator for 24 h. According to the grouping, the blank group, miR-8485 NC, and miR-8485 mimic were detected. In addition to the blank group, the BJOE group and BJOE + 3-MA group were added to another 6-well plate. The original medium of the 6-well plate was sucked out, washed with PBS, added the prepared Monosulfonylcadaveramide (MDC) staining solution, and then incubated at 37°C for 15 min. After sucked out, PBS was washed again, and then, the dyeing solution was washed with PBS. The basic medium was added to re-incubate for 15 min. The fluorescence microscope was observed, counted, and photographed.

### Statistical Analysis

The statistical analysis in this study is carried out by using Graph Pad Prism 9.3. The above experimental results are obtained by more than three independent repetitive operations, and all experimental data are expressed as mean ± standard deviation. The statistical test level is *α* = 0.05, and *p* < 0.05 indicated that the difference is statistically significant. *p* < 0.001 and below indicated that the difference is statistically significant.

## Results

### Components Analysis of *B. javanica* Oil Emulsion

**FIGURE 1:**
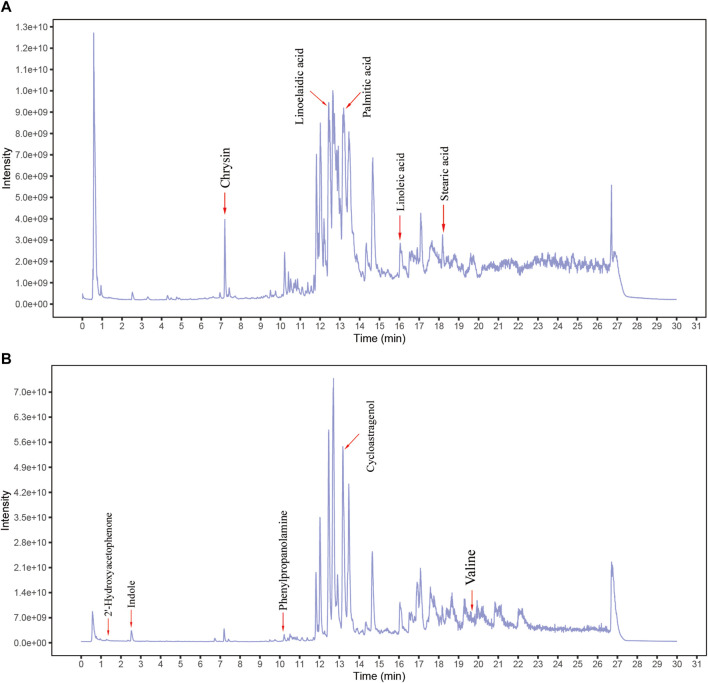
Identification of chemical components of Brucea javanica oil emulsion (BJOE). BJOE samples were examined by UHPLC‐MS/MS. Total ion chromatography in negative **(A)** and positive **(B)** ion modes for BJOE samples are shown.

After the analysis of BJOE samples by UHPLC-MS/MS, researchers determined that 207 kinds of main chemical constituents were found (Figures 1A,B). The main species include 56 Terpenoids, 25 Flavonoids, 20 Alkaloids, 8 Aliphatic acyls, 7 Phenylpropanoids, five fatty acids and amino acid derivatives, and 81 other species. The main compounds include Chrysin, Linoelaidic acid, Linoleic acid, Palmitic acid, Stearic acid, 2′-Hydroxyacetophenone, Cycloastragenol, indole, phenylpropanolamine, and Valine.

### *B. javanica* Oil Emulsion Increases miR-8485 Expression in SKOV3 Cell

**FIGURE 2:**
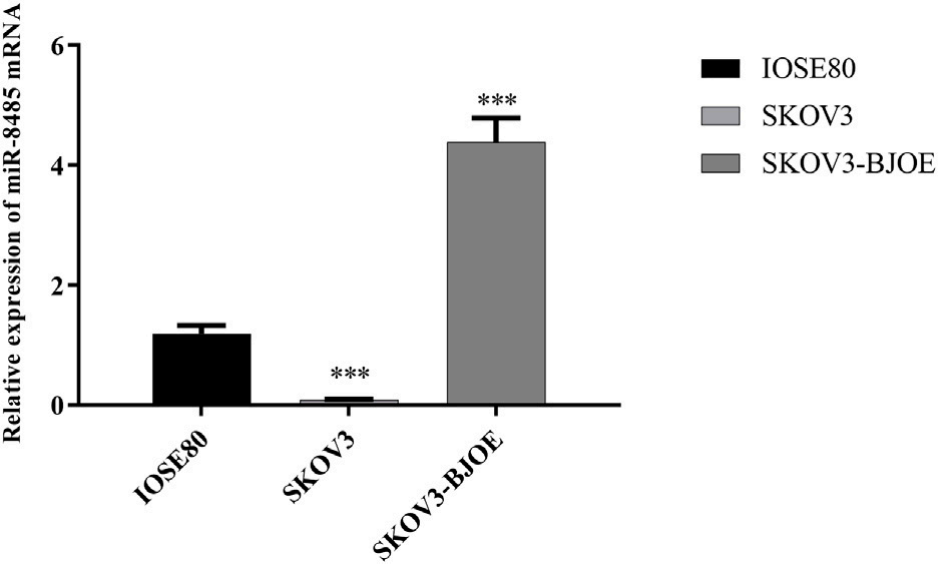
Expression of miR-8485 in different ovarian cells. (SKOV3 group was compared with IOSE80 group (n = 3). Data were shown as mean ± SD, ***p < 0.001; SKOV3 group was compared with SKOV3-BJOE group (n = 3). Data were shown as mean ± SD, ***p < 0.001).

To explore the effect of BJOE on miR-8485 expression, SKOV3 cells were treated with BJOE for 24 h. The qRT-PCR results showed that the expression of miR-8485 in the SKOV3 cell was significantly decreased compared with that in IOSE80 cells, whereas its expression was up-regulated after BJOE treatment in the SKOV3 cell (Figure 2). This finding suggests that BJOE contributes to induction of miR-8485 expression in SKOV3 cells.

### *B. javanica* Oil Emulsion and Overexpression of miR-8485 Inhibit the Proliferation of SKOV3 Cell

**FIGURE 3:**
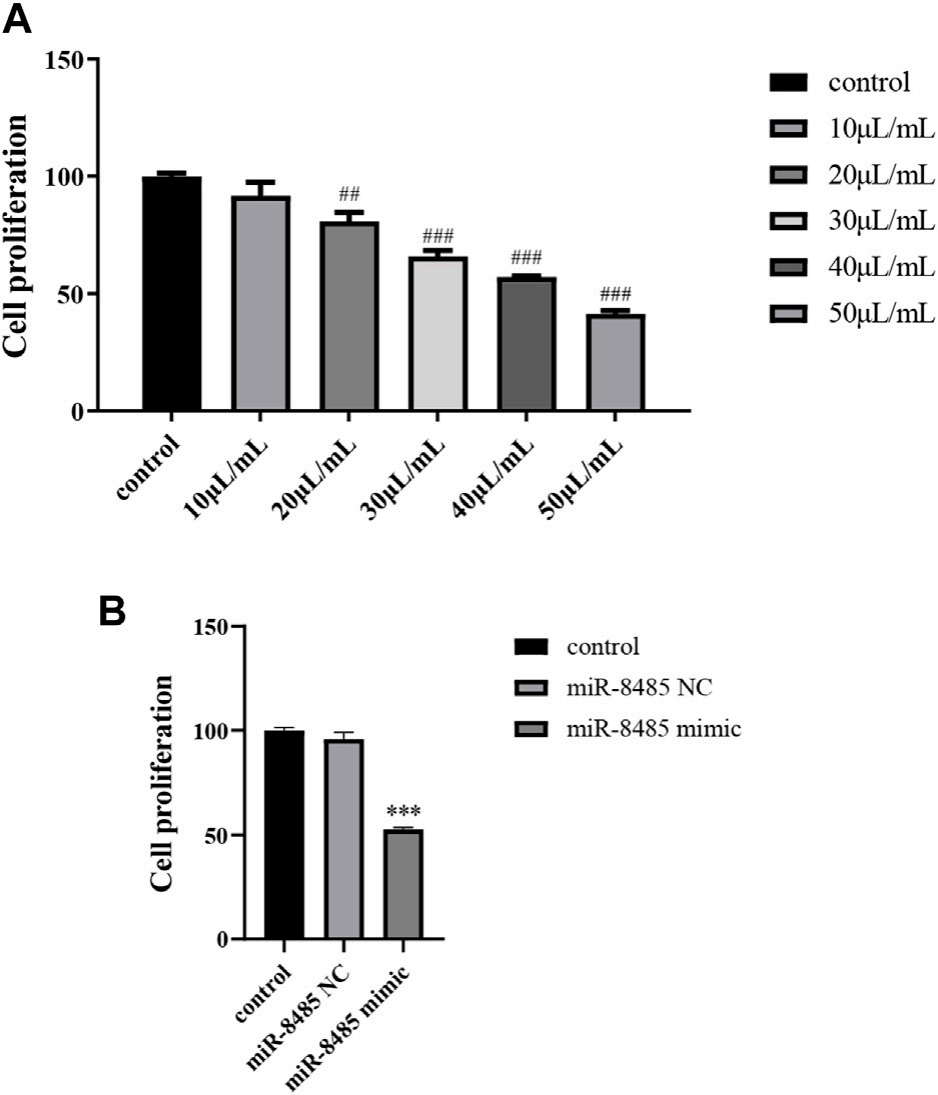
**(A)** Effects of different concentrations of drugs on cell proliferation; **(B)** overexpression of miR-8485 on cell proliferation. (All the *p* values were compared with the control group (n = 3). Data were shown as mean ± SD, ^##^ *p* < 0.01, ^###^ *p* < 0.001, ***p < 0.001).

Next, the impact of BJOE and miR-8485 mimic on SKOV3 cell proliferation were explored using the CCK8 experiment. As shown in Figures 3A,B, compared with the control group, with the increase of the drug concentration, the inhibitory effect of BJOE on the proliferation of SKOV3 cell gradually increased, and the absorbance value decreased significantly. Also, the IC50 at 24 h was 44.37 μl/ml. Since the 50 μl/ml concentration significantly decreased the cell proliferation activity, we selected 50 μl/ml for further mechanism exploration (*p* < 0.05). Similar to this, miR-8485 overexpression also inhibited the proliferation of SKOV3 cells.

### *B. javanica* Oil Emulsion and Overexpression of miR-8485 Induce SKOV3 Cell Apoptosis

**FIGURE 4:**
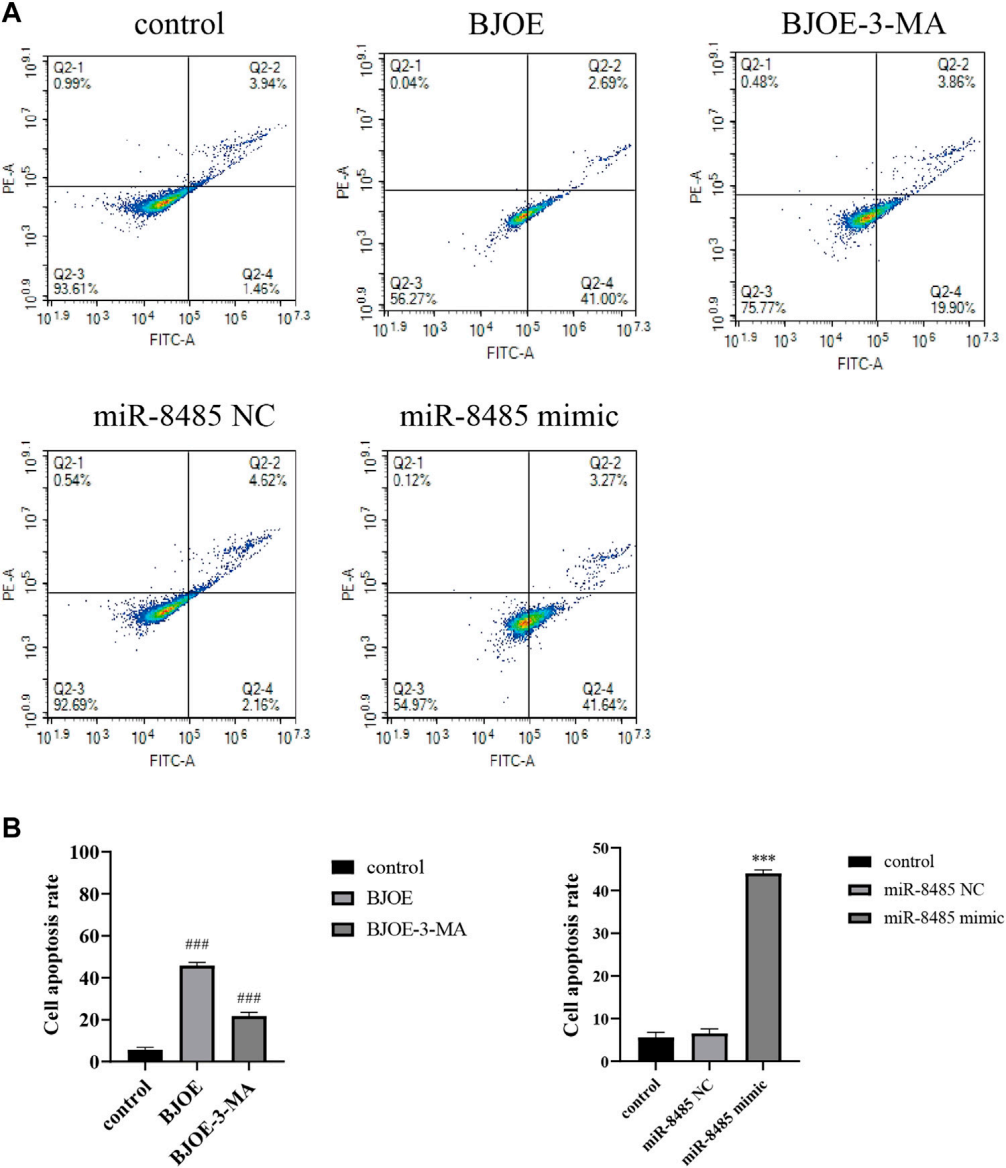
Effects of Brucea javanica oil emulsion and overexpression of miR-8485 on apoptosis of SKOV3 cell. (All the *p* values were compared with the control group (n = 3). Results are represented as mean ± SD. ^###^ *p* < 0.001, ***p < 0.001).

The effects of BJOE and miR-8485 overexpression on SKOV3 cell apoptosis were observed by flow cytometry. By using AnnexinVFITC/PI staining, it was found that as shown in Figures 4A,B, the apoptosis of BJOE group was significantly increased compared with that of the blank group, reaching 43.69%, while after adding the 3-MA inhibitor, the apoptosis rate in BJOE + 3-MA group was 23.76%. As illustrated in Figures 4A,B, the apoptosis rate of SKOV3 cells overexpressing miR-8485 was significantly increased by 44.91% compared with the blank group and miR-8485-NC group. As a whole, these observations indicate that BJOE and miR-8485 overexpression can promote the apoptosis of SKOV3 cells.

### *B. javanica* Oil Emulsion and Overexpression of miR-8485 Stimulate SKOV3 Cell Autophagy

**FIGURE 5:**
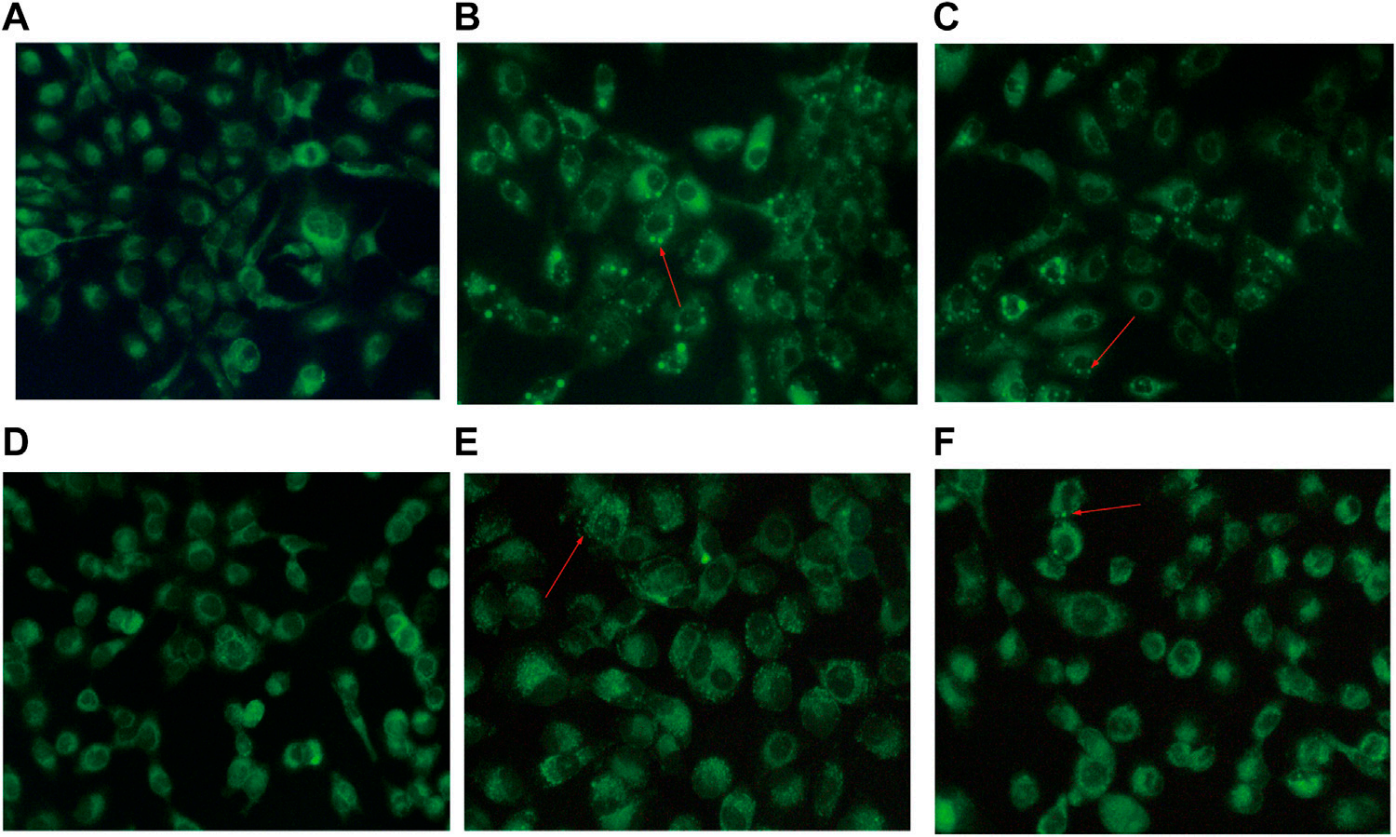
Effects of drugs and miR-8485 on autophagy. [**(A)** control, **(B)** BJOE, **(C)** BJOE + 3-MA, **(D)** miR-8485 NC, **(E)** miR-8485 mimic, **(F)** miR-8485 mimic + 3-MA. The red arrow indicates autophagy vesicles].

The effect of BJOE on autophagy vesicles was observed by MDC fluorescence staining in SKOV3 cells. As shown in Figures 5A–C, the autophagic vesicles in BJOE group were significantly increased compared with those in the blank group, while the autophagic vesicles were decreased after treatment with 3-MA, indicating that BJOE exerts a promotive effect on SKOV3 cell autophagy. Similarly, as shown in Figures 5D–F, a similar effect was observed in SKOV3 cells overexpressing miR-8485.

### Identification of LAMTOR3 as a Direct Target of miR-8485

**FIGURE 6:**
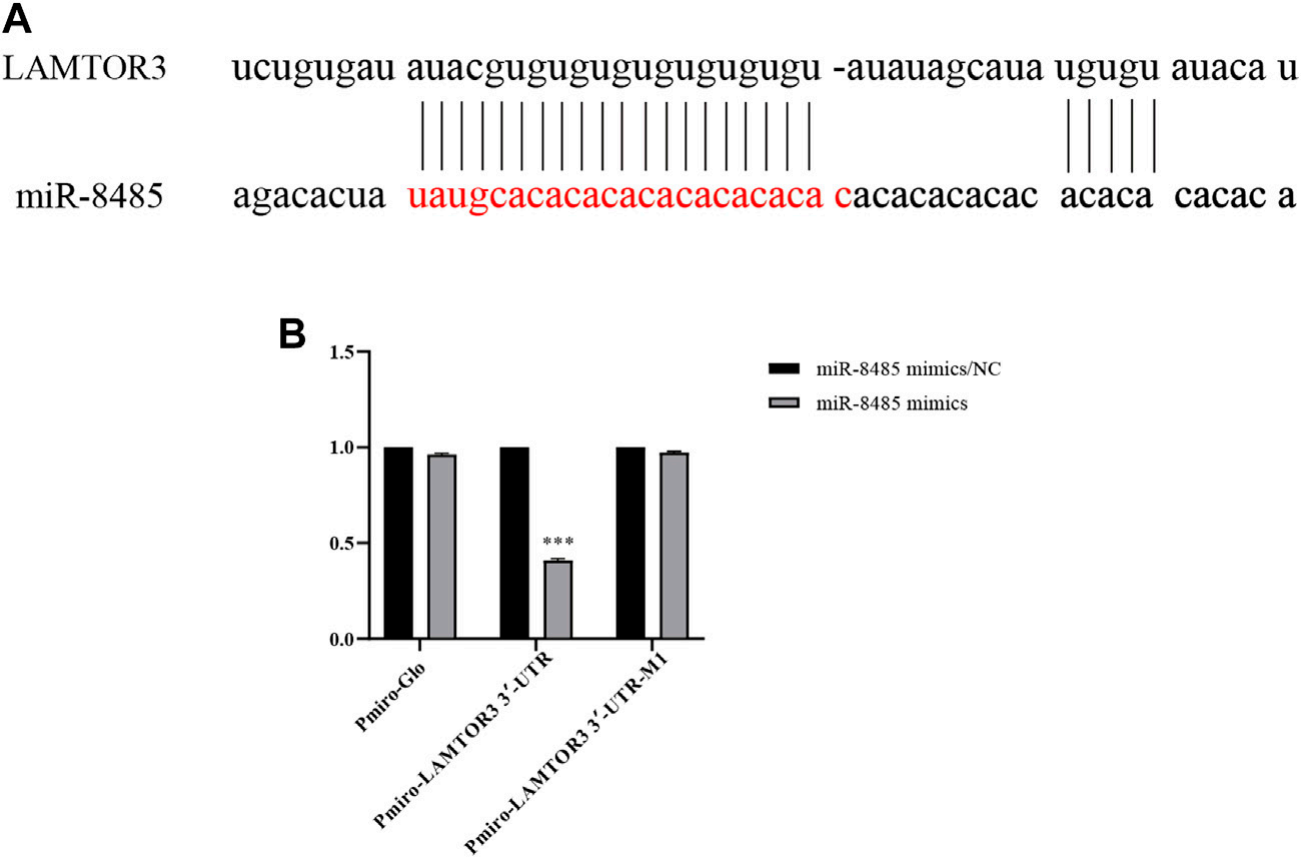
LAMTOR3 is a target of miR-8485. [**(A)** Prediction of relationship between miR8485 and LAMTOR3 by biological information. **(B)** The targeted relationship between miR-8485 and LAMTOR3 was confirmed by luciferase assay (n = 3). MiR-8485 mimics group was compared with miR-8485 mimics/ NC. Data were shown as mean ± SD, ***p < 0.001].

The potential targets of miR-8485 were predicted by miRDB, Target Scan, and miRDB databases. A binding site was found between miR-8485 and LAMTOR3 (Figure 6A). Next, subsequently, the wild type (Wt) or mutant (Mut) LAMTOR3 fragment was linked to pmirGLO vector, which were co-transfected into SKOV3 cells with miR-8485 mimic or mimic control. Luciferase reporter assay revealed that co-transfection of LAMTOR3-WT with miR-8485 mimic led to a significant decrease in the luciferase activity, while this effect disappeared when the miR-8485 binding site was mutated (Figure 6B). In summary, miR-8485 can directly target LAMTOR3.

### Effect of *B. javanica* Oil Emulsion and miR-8485 Overexpression on LAMTOR3, mTOR, ATG13, and LC3II Expression in SKOV3 Cells

**FIGURE 7:**
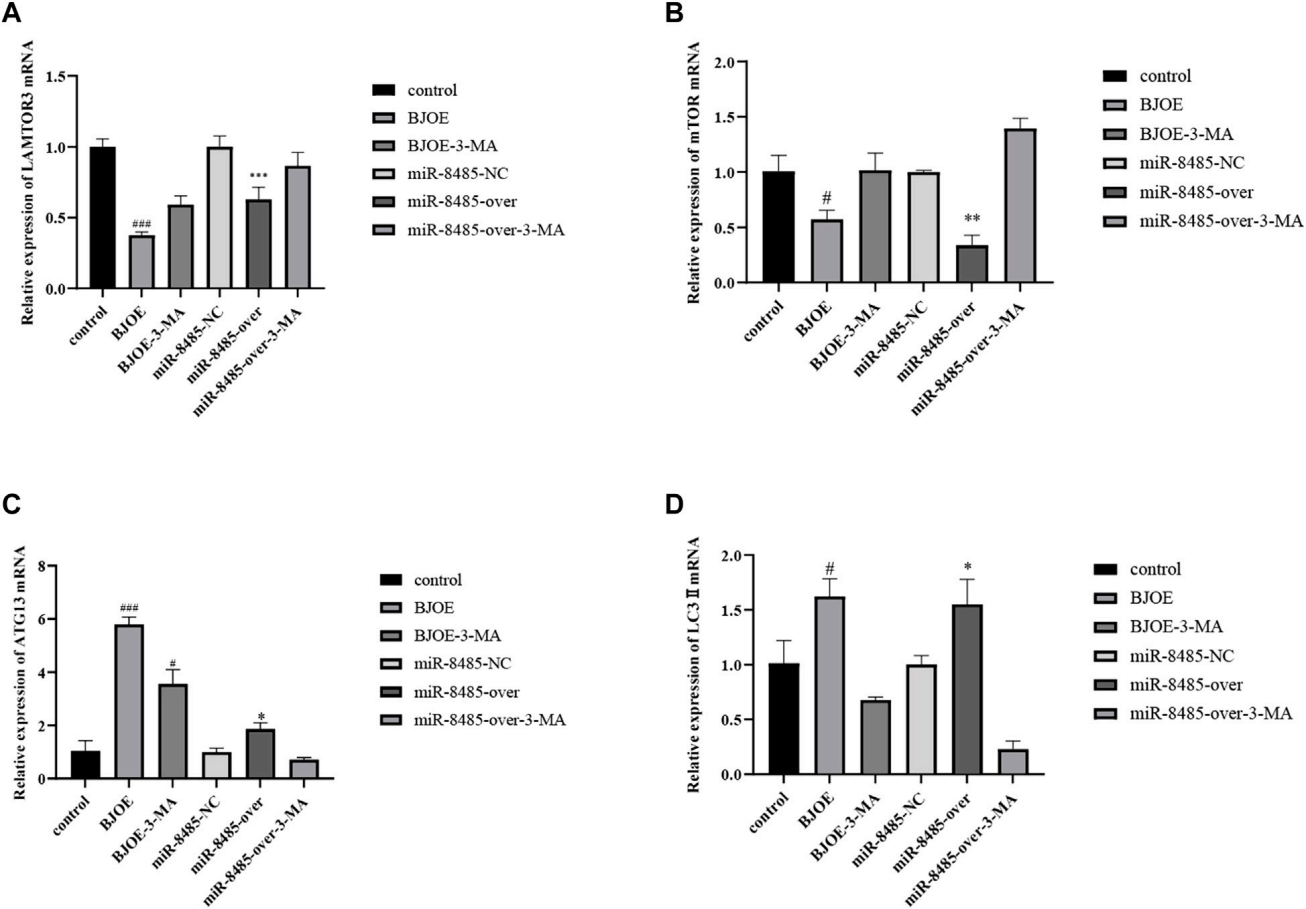
Effects of BJOE and overexpressed miR-8485 on autophagy gene mRNA. [**A–D** Effect of BJOE, overexpression of miR-8485 and autophagy inhibitor on relative mRNA expression of LAMTOR3, mTOR, ATG13 and LC3II, All the *p* values were compared with the control. All the *p* values were compared with the control group (n = 3). Data were shown as mean ± SD, ^#^ *p* < 0.05, ^###^ *p* < 0.001; *p < 0.05, **p < 0.01, *** *p* < 0.001].

**FIGURE 8:**
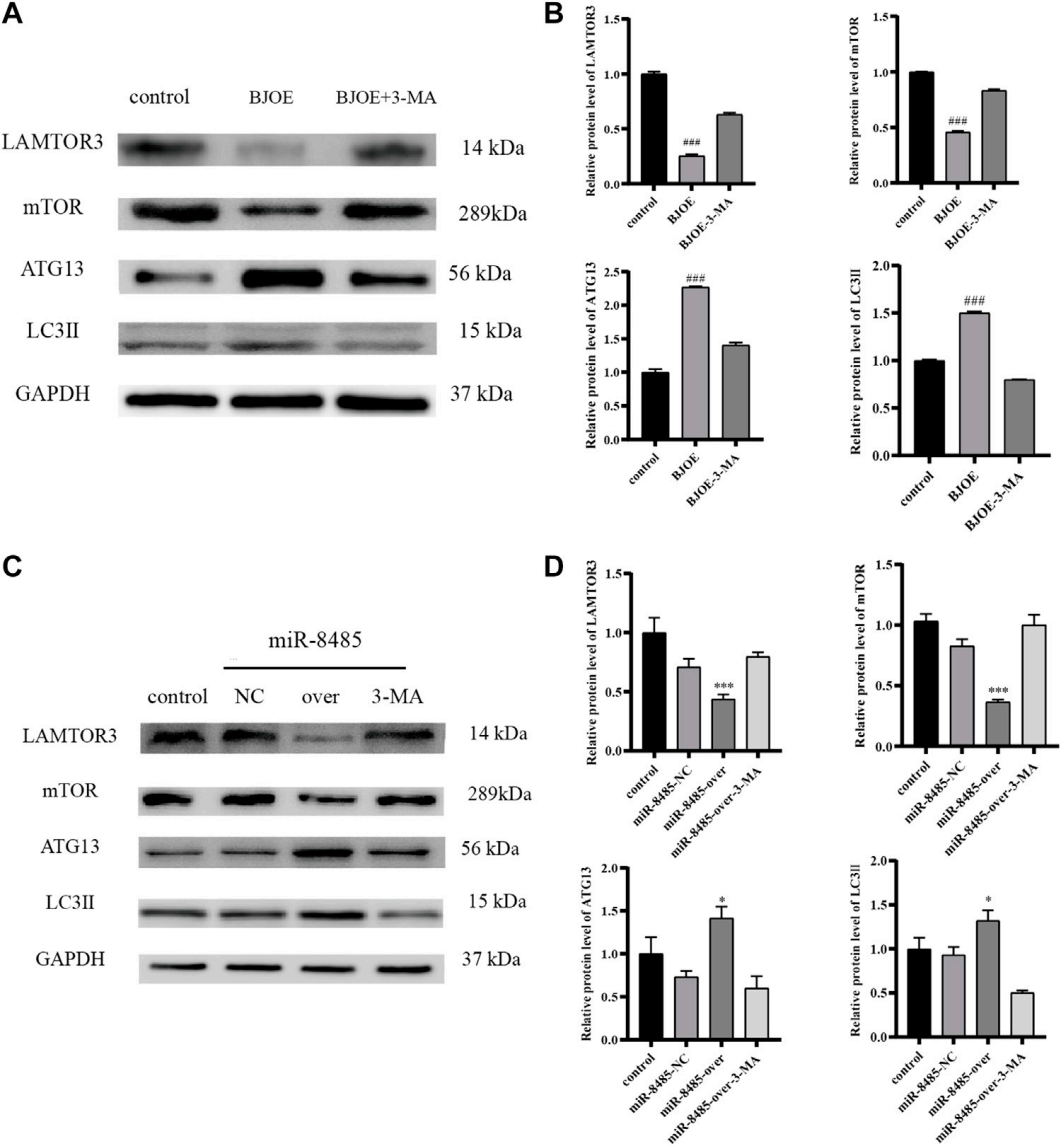
Effects of BJOE and miR-8485 overexpression on autophagy-related gene proteins [**(A,B)** Western blot analysis of protein expression of LAMTOR3, mTOR, ATG13 and LC3II in control group, BJOE and BJOE inhibitor group. **(C,D)** Western blot analysis of protein expression of LAMTOR3, mTOR, ATG13 and LC3II in control group, miR-8485 NC group, miR-8485 overexpression group and miR-8485 overexpression inhibitor group. All the *p* values were compared with the control group (n = 3). Data were shown as mean ± SD, ^###^ *p* < 0.001; *p < 0.05, *** *p* < 0.001].

According to the results of qRT-PCR, as shown in Figures 7A–D, BJOE and miR-8485 overexpression can lead to changes in autophagy-related genes in SKOV3 cells. Compared with the blank group, the mRNA levels of LAMTOR3 and mTOR genes in the BJOE group and miR-8485-over group were significantly decreased, and the mRNA levels of ATG13 and LC3 II genes were significantly increased. However, after addition of 3-MA inhibitor in these two groups, the changes of gene expression were consistent with the expression trend of the blank group. This indicates that BJOE and overexpression of miR-8485 could promote autophagy in SKOV3 cells and significantly inhibit cell proliferation (*p* < 0.05). The results of Western blot experiment, as shown in Figures 8A–D, showed that BJOE and overexpressed miR-8485 downregulated LAMTOR3 protein expression, inhibited mTOR protein phosphorylation, and increased ATG13 and LC3II protein expression levels, which was consistent with the results of qRT-PCR experiment, suggesting that BJOE promotes autophagy of SKOV3 cells, which may be involved in the regulation of the LAMTOR3/mTOR/ATG13 signal axis by miR-8485.

## Discussion

The research on the effect of traditional Chinese medicine on autophagy of ovarian cancer is in its infancy, and few literatures to explore the related role and mechanism exist, so further in-depth study is very important. The decrease of autophagy in ovarian cancer cells can promote cell proliferation and migration, thereby affecting the occurrence and development of ovarian cancer. Studies found that BJOE can affect and regulate the autophagy level of cells, but the specific role and mechanism are unclear (Yan et al., 2015; Zhu and Pan, 2018). Next, BJOE regulates autophagy pathway in ovarian cancer has not been reported.

This experiment showed that BJOE significantly increased autophagy and caused significant changes in related factors in mTOR signal pathway compared with ovarian cancer SKOV3. mTOR is a key target in multiple autophagy pathways, and recent studies showed that traditional Chinese medicine can regulate the proliferation, apoptosis, and multidrug resistance of ovarian cancer cells by affecting mTOR-related signaling pathways (Gao et al., 2022; Li et al., 2022; Yang et al., 2022; Zhu et al., 2022). Mammalian rapamycin target protein activator 3 (LAMTOR3), alias MP1, is located on human chromosome 4q22.3 (Sun and Xu, 2012). It is a protein-coding gene and encodes scaffold protein, which is one of the Ragulator complexes (Sun and Xu, 2012). It binds to lysosomes and activates Rag GTPases through lipid modification and anchoring and recruits mTORC1 until lysosomes are activated (Sun and Xu, 2012). The activated mTORC1 can simultaneously pity and acidify the homologous proteins ULK1 of ATG13 and ATG1 in mammals to inhibit autophagy (Sun and Xu, 2012). ATG 13 is involved in the formation of ULK1 complex, which is an important target for autophagy regulation (Alers et al., 2011; Wallot-Hieke et al., 2018). The increase of autophagy promotes the transformation of LC3-I to LC3-II, and the increase of LC3-II is positively correlated with the level of autophagy in cells, so it can be used as a specific marker protein of autophagy as an indicator to evaluate the degree of autophagy.

MicroRNAs (miRNAs) are a class of endogenous noncoding RNAs with regulatory functions found in eukaryotes c. Most miRNAs are differentially expressed in normal tissues and blood compared with blood and cancerous tissues of tumor patients (Bi et al., 2021; Dai et al., 2021). Next, the main function of miRNAs is to regulate gene expression in organisms by degrading or inhibiting the translation of target mRNAs (Bi et al., 2021). Studies showed that miRNAs affect cell proliferation, invasion and drug resistance by regulating autophagy (Cai et al., 2021; Zhang et al., 2021bib_Zhang_et_al_2021). The mechanism of miR-8485 in ovarian cancer has not been reported. The miR-8485 detected by dbDEMC (Human Cancer differential expression MiRNAs Database) shows a tendency of low expression in the blood of different cancers, such as ovarian cancer.

In this study, the qRT-PCR experiment showed that the expression level of miR-8485 in SKOV3 cells was significantly lower than that in IOSE80 cells, but the expression level of SKOV3 cells was increased after BJOE treatment. Then, we confirmed, by CCK8 and flow cytometry, that BJOE and overexpression of miR-8485 could inhibit the proliferation of SKOV3 cell and increase cell apoptosis. Further experiments with MDC staining showed that autophagy vesicles were significantly increased after treatment with BJOE and overexpression of miR-8485 in SKOV3 cell. By increasing autophagy of SKOV3 cell, BJOE and overexpression of miR-8485 may inhibit cell proliferation and induce cell apoptosis. In previous experiments, we found that the pathways of *B. javanica (L.) Merr.* [Simaroubaceae] in ovarian cancer cells were mainly concentrated in mTOR, VEGF, and MAPK through metabonomics and bioinformatics analysis. Thus, based on the previous research and experimental progress, we mainly carried out more in-depth research on mTOR pathway. Afterward, by using qRT-PCR and Western Blot experiments, we further found that the expression of autophagy-related genes LAMTOR3 and mTOR was inhibited by BJOE and overexpression of miR-8485 in SKOV3 cell, thereby increasing the expression of ATG13 and LC3 II, confirming that BJOE and overexpression of miR-8485 could increase autophagy in SKOV3 cells. Further biosignaling analysis showed that miR-8485 demonstrated binding sites with LAMTOR3 mRNA, indicating that miR-8485 could directly control the expression of LAMTOR3. At the same time, the changes of gene expression in BJOE group and miR-8485 overexpression group were reversed by 3-MA inhibitor, and the changes of gene expression were opposite to the BJOE group and overexpression group, indicating that BJOE group and overexpression of miR-8485 can increase autophagy and inhibit cell proliferation in SKOV3 cells.

## Conclusion

At present, BJOE has been clinically proved to be effective as an adjuvant therapy for various tumors. However, the specific mechanism is unknown, which affects its wide application in clinical practice, especially in ovarian cancer. This study found that BJOE may affect the expression of LAMTOR3 to inhibit the increase of mTOR and negatively regulate the expression of ATG13 by upregulaing miR-8485 expression. Then, the autophagy incidence was increased, and cell proliferation was inhibited in SKOV3 cell. MiR-8485 may be a key factor in BJOE promoting autophagy and apoptosis and inhibiting cell proliferation in ovarian cancer cells.

## Data Availability

The original contributions presented in the study are included in the article/Supplementary Material, and further inquiries can be directed to the corresponding author.

## Abbreviations

BJOE, *B. javanica* Oil Emulsion; mTOR, mammalian rapamycin target protein; MDC, Mono-dansylcadaverine; LAMTOR3, mammalian rapamycin target protein activator 3; ATG13, autophagy-related 13; LC3-II, microtubule associated protein 1 light chain 3 beta; PI3K, Phosphatidylinositol 3-kinase; AKT, Serine/threonine kinase 1; ULK1, autophagy activating kinase 1; ATG1, autophagy-related 1; VEGF, vascular endothelial growth factor; MAPK, mitogen activated kinase-like protein; NRXN1, human neurofilament 1; and TDP-43, TAR DNA binding protein.
