# Supplementary figures and images for: Brucea javanica Oil Emulsion Promotes Autophagy in Ovarian Cancer Cells Through the miR-8485/LAMTOR3/mTOR/ATG13 Signaling Axis
Source: Front Pharmacol. 2022 Jul 25;13:935155. doi: 10.3389/fphar.2022.935155 (PMC9358144; doi:10.3389/fphar.2022.935155)

— 1

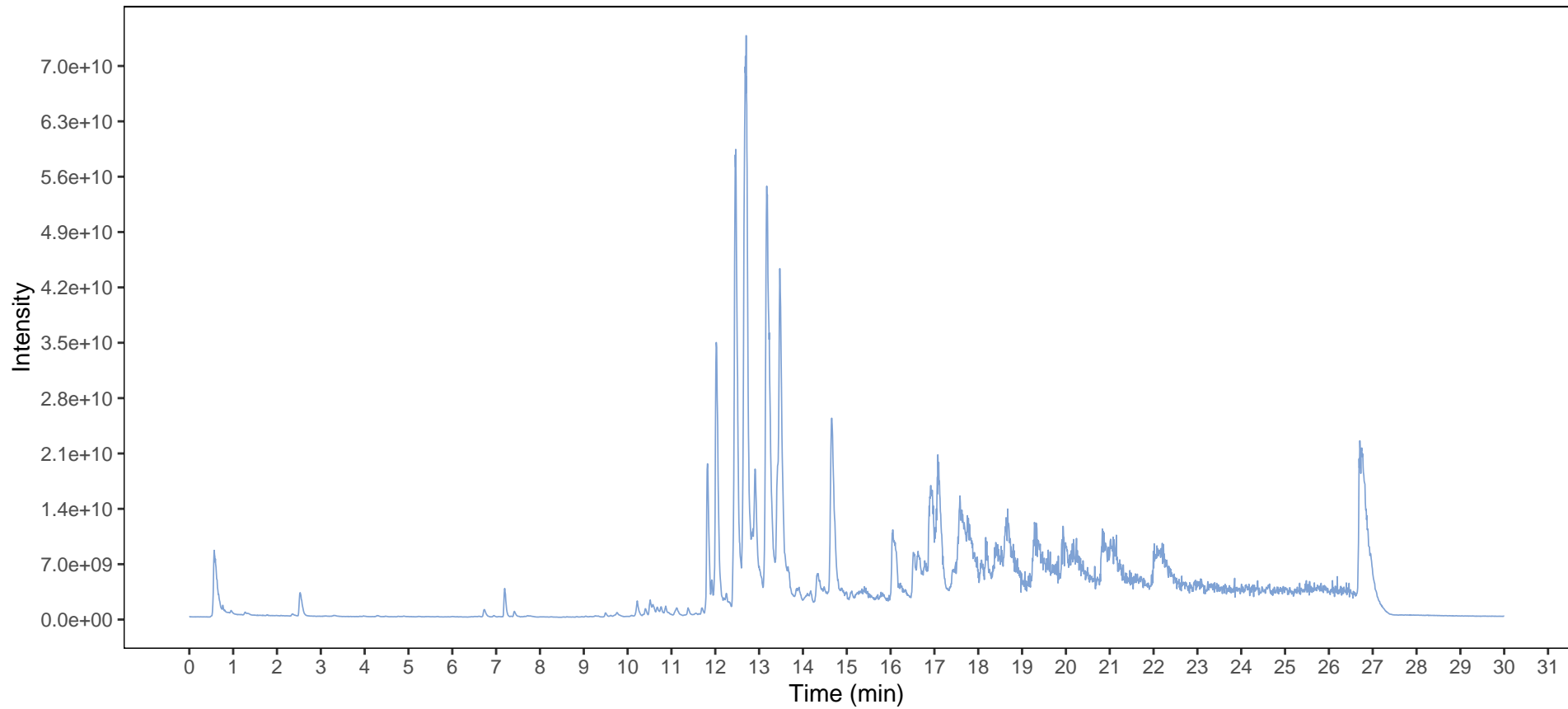

Supplement: Supplementary file 1 [file DataSheet2.PDF]

The full uncropped and unedited versions of the Western blots:

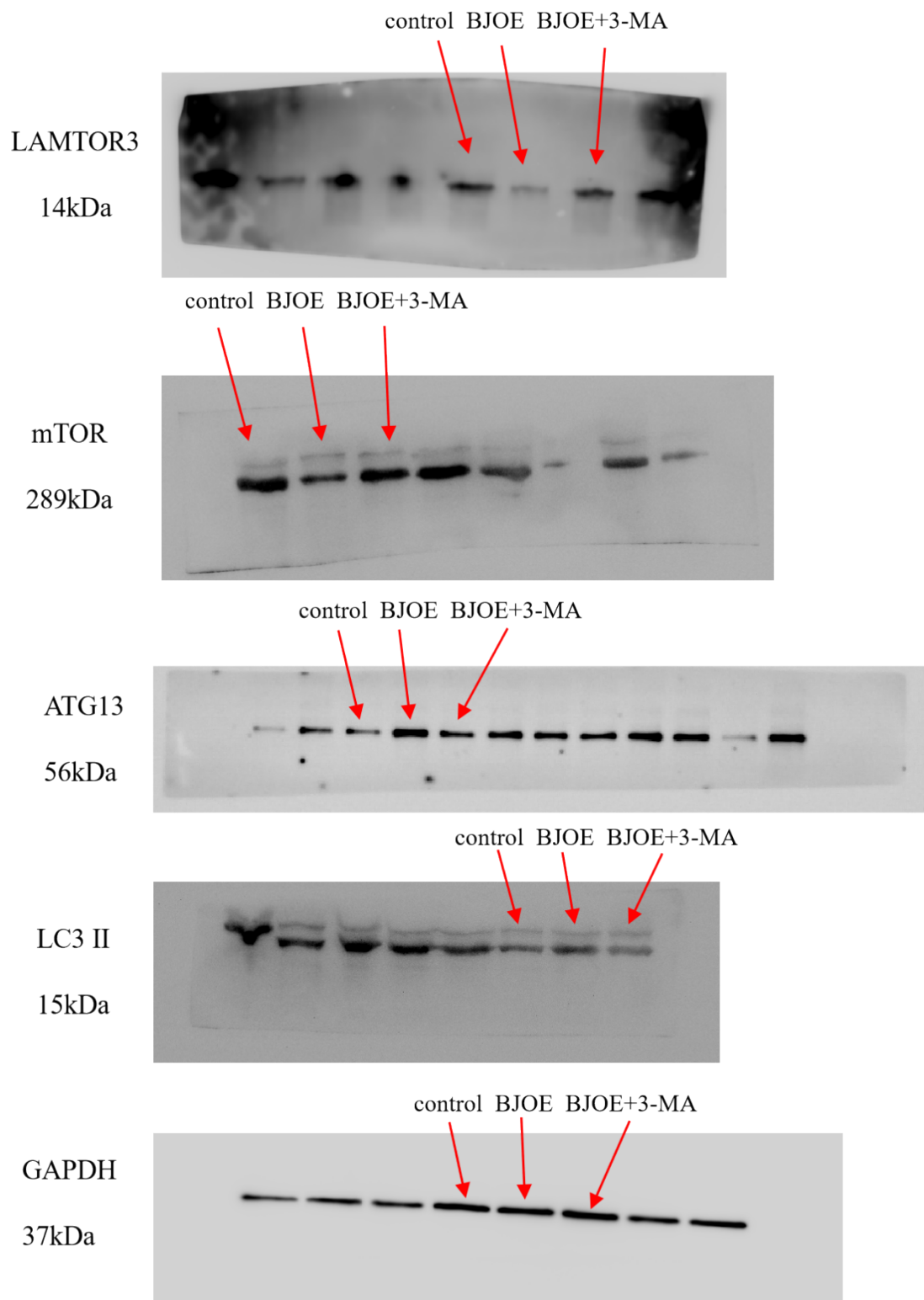

Fig. 1

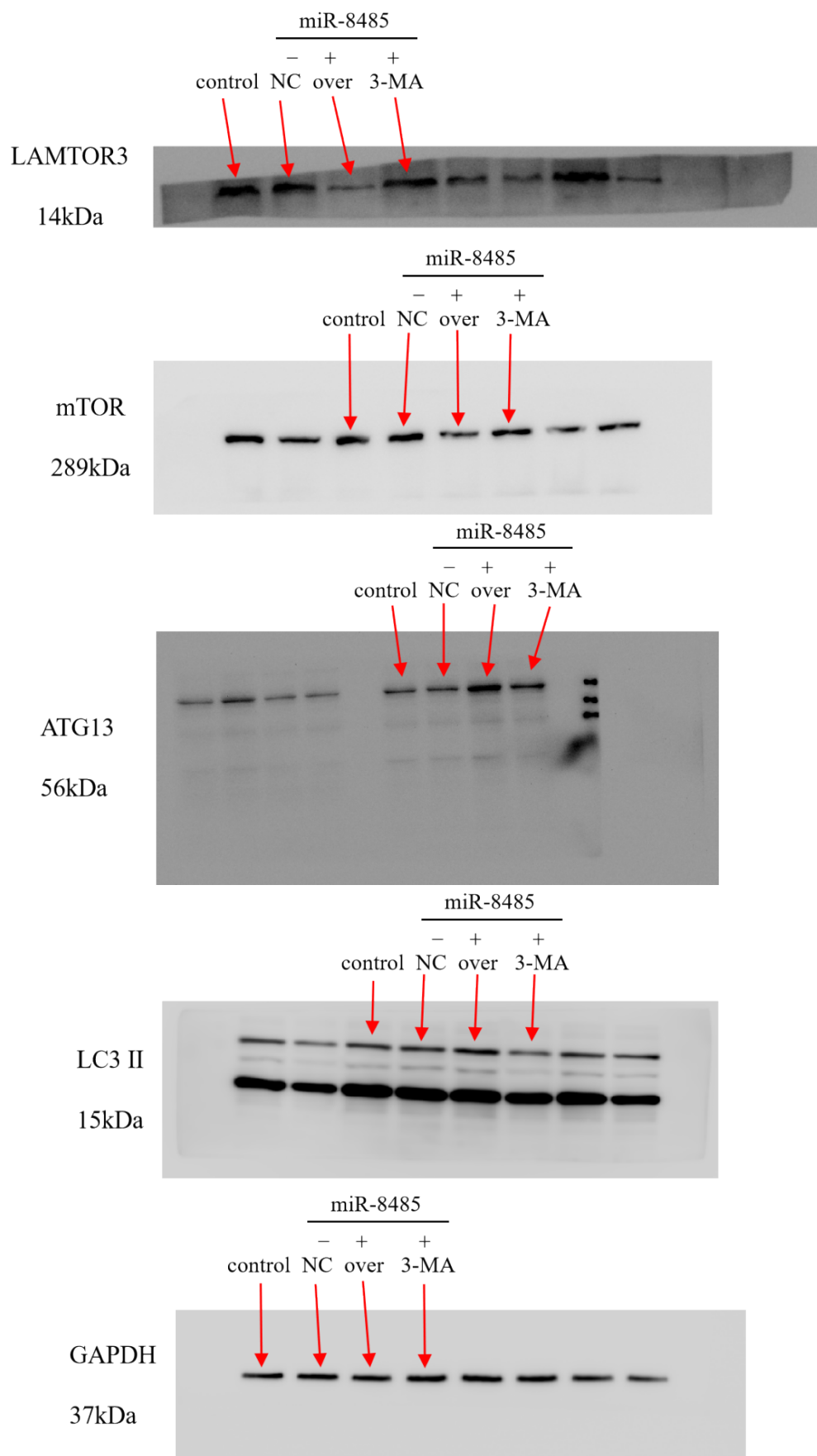

Fig. 2

Supplement: Supplementary file 2 [file DataSheet3.PDF]

— 1

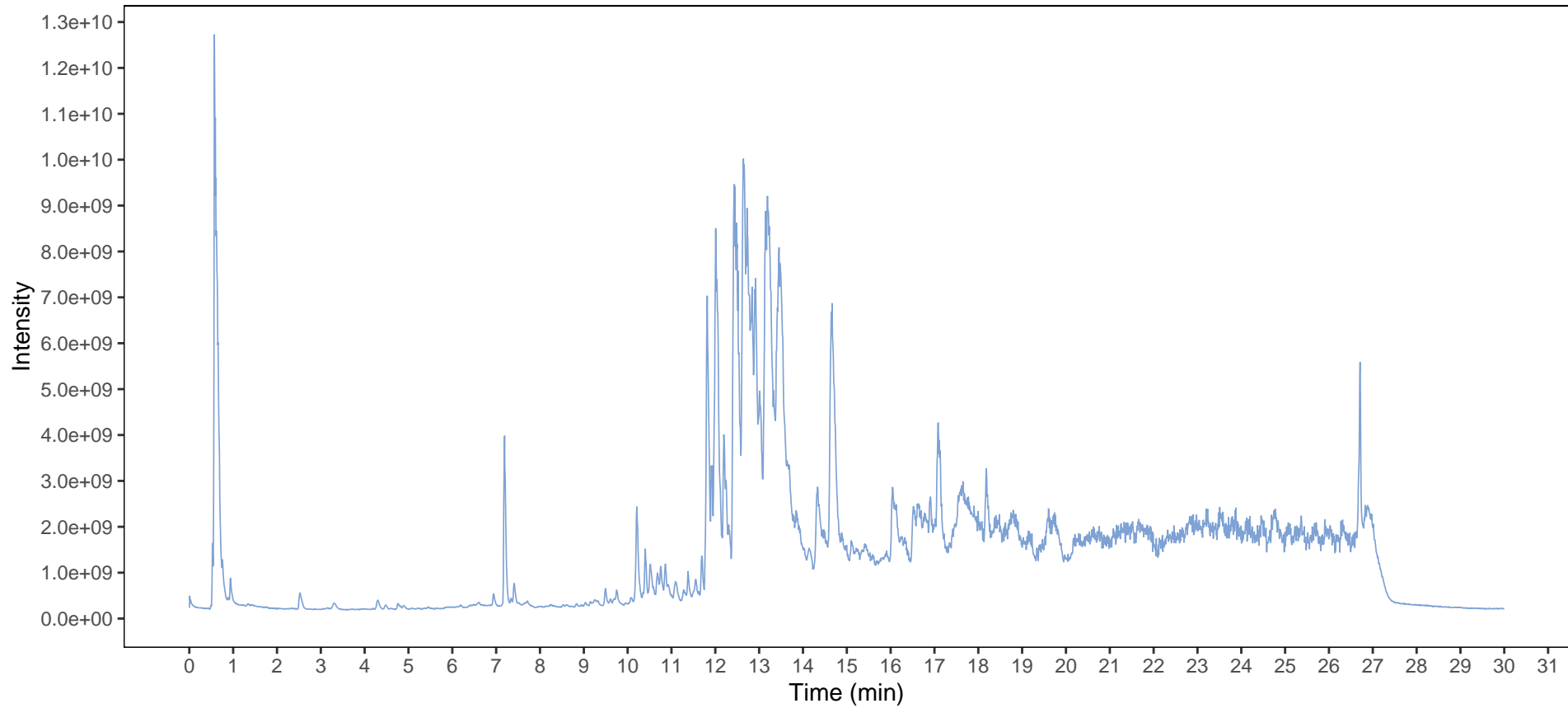

Supplement: Supplementary file 3 [file DataSheet1.PDF]
